# St. Bartholomew's Hospital: Reports on Patients

**Published:** 1915-04-24

**Authors:** 


### ST. BARTHOLOMEW'S HOSPITAL: REPORTS ON PATIENTS.

### Text of the Rules for the Medical Staff.

We welcome with much satisfaction the signs of alertness characterising the action of the treasurer and almoners, and also of the governors, of St. Bartholomew's Hospital. For many months hospitals have questioned what course it is wisest to adopt in regard to reports upon patients by members of the junior medical staff and the payment of fees to them for their services. The following regulations cannot fail to excite general interest, and may prove suggestive to other hospitals. We have dealt fully with the questions raised in an article on page 87.

### ST. BARTHOLOMEW'S HOSPITAL.

#### Regulations as to Reports upon Patients and Acceptance of Fees for same.

Members of the junior medical staff are informed :

(*a*) That the records relating to patients—*i.e.*, the notes of the cases and all information obtained in examination or otherwise—must be regarded as confidential.

(*b*) The reports upon a patient must not be furnished, or inspection of the records of a case allowed without the written consent, or on the written application, of the patient or his authorised representative.

(*c*) That in the event of a member of the junior medical staff being applied to for a report on a case, he is to refer the matter to the clerk of the hospital, who will see that the application is properly authorised.

(*d*) That the service of a subpœna only requires the person to give evidence in Court, and does not authorise the giving of information to the person serving the subpœna.

(*e*) That a patient in the hospital must not be subjected to examination by a medical practitioner, or other person, acting on behalf of any outside body, without the consent of the patient, and, before such consent is obtained, the patient should be fully informed by an authorised official of the hospital as to the object of such examination. This examination should be made in the presence of members of the junior medical staff.

Subject to the foregoing the treasurer and almoners do not object to members of the junior medical staff of the hospital asking for and receiving payment for written reports given by them regarding the condition of patients who are, or have been, under their care, when such reports are required for the furtherance of pecuniary interests, such as claims for money, legal proceedings, and the like, but only when the same involve an expression of professional opinion on the case, and do not consist merely of statements of fact. Except under these conditions, or for evidence in a Court of Justice, a demand or acceptance of any fee or payment in relation to a hospital patient is strictly prohibited.

For any such report a medical officer is forbidden to demand a fee exceeding one guinea.

Adopted by the Treasurer and Almoners.
March 18, 1915.

---

#### Form of Request and Authority for a Report upon a Patient of St. Bartholomew's Hospital.

[I or We] ..............................................................
of ..............................................................
*(acting on behalf of) ..............................................
* Strike out these words if they do not apply.
......................request that a Report may be furnished to [me or us] ..................with reference to ................. ........................who [is or was in] .....................under treatment as an in-patient [out-patient] at St. Bartholomew's Hospital.

The following are the special points upon which information is required, viz. :—

..............................................................
Date .................. Signature..................................

---

#### Form to be Filled in and Signed by the Patient before a Report can be Given.

To the Governors of St. Bartholomew's Hospital.
I ..............................................................
of ..............................................................
consent to a Report upon my case being furnished to ............................................... as above requested.
Date .................. Signature ..................................

---

St. Bartholomew's Hospital, London, E.C.
..........................191 .

Dear Sir,—

I have to acknowledge your letter of the ......... instant asking for a Report upon the case of.................................. .........................................(formerly) a patient of this Hospital, and subject to the accompanying form being returned to me duly filled up, together with a cheque for the Medical Officer's fee of One Guinea, a Report will be sent you.

I am, Dear Sir,
Yours faithfully,
..............................
Encl. Clerk to the Governors.

